# Complex Suicide Involving Hanging and Self-Inflicted Incision: A Case Report

**DOI:** 10.7759/cureus.63514

**Published:** 2024-06-30

**Authors:** Arjun T, Venkatesan M, Priyadarshee Pradhan

**Affiliations:** 1 Forensic Medicine and Toxicology, Sri Ramachandra Institute of Higher Education and Research, Chennai, IND; 2 Forensic Medicine, Sri Ramachandra Institute of Higher Education and Research, Chennai, IND

**Keywords:** ligature mark, incised wound, suicide, hanging, complex suicide

## Abstract

Suicide, the act or instance of voluntarily and intentionally taking one’s own life, is a leading global cause of death. Complex suicide involves the use of more than one method and can be either planned or unplanned. Frequently seen methods include the ingestion of a toxic substance in combination with hanging, the use of firearms with hanging, drowning combined with injuries from knives, and jumping from heights. We present a case of complex suicide in which the victim used hanging and a self-inflicted incision wound on the wrist. This case highlights the significance of a detailed medicolegal death investigation, including interviews with relatives and a thorough forensic autopsy to ascertain the cause and manner of death.

## Introduction

Suicide is the act of taking one’s own life voluntarily and intentionally. In India, an analysis of data over five years revealed that the most common methods of suicide were hanging (51.5%) and poisoning (26.7%) according to the National Crime Records Bureau (NCRB, 2018). Men were the predominant victims, with the highest incidence among those aged 18-30 years (34.9%), followed by those aged 31-45 years (31.6%) [1]. There are two types of suicide: simple and complex. Scholars have defined complex suicide as the adoption of more than one method to ensure death by suicide [1]. Complex suicide is either planned or unplanned [2]. “Primary complex suicide” means the victim uses several methods simultaneously, whereas “secondary complex suicide” means the victim uses an alternative second method only after the first method fails [2]. Victims can combine many methods, such as drowning, poisoning, discharging a firearm, and jumping from heights, in a complex suicide. In unplanned complex suicide, if the first method fails, the victim may choose another method [3]. According to the literature, complex suicide accounts for 1.5%-5% of all suicides [1]. The adoption of more than one method for suicide may create doubt and confusion about whether it is homicide or suicide. In this paper, we report a case of complex suicide by a self-inflicted incision wound on the wrist combined with hanging.

## Case presentation

**Figure 1:**
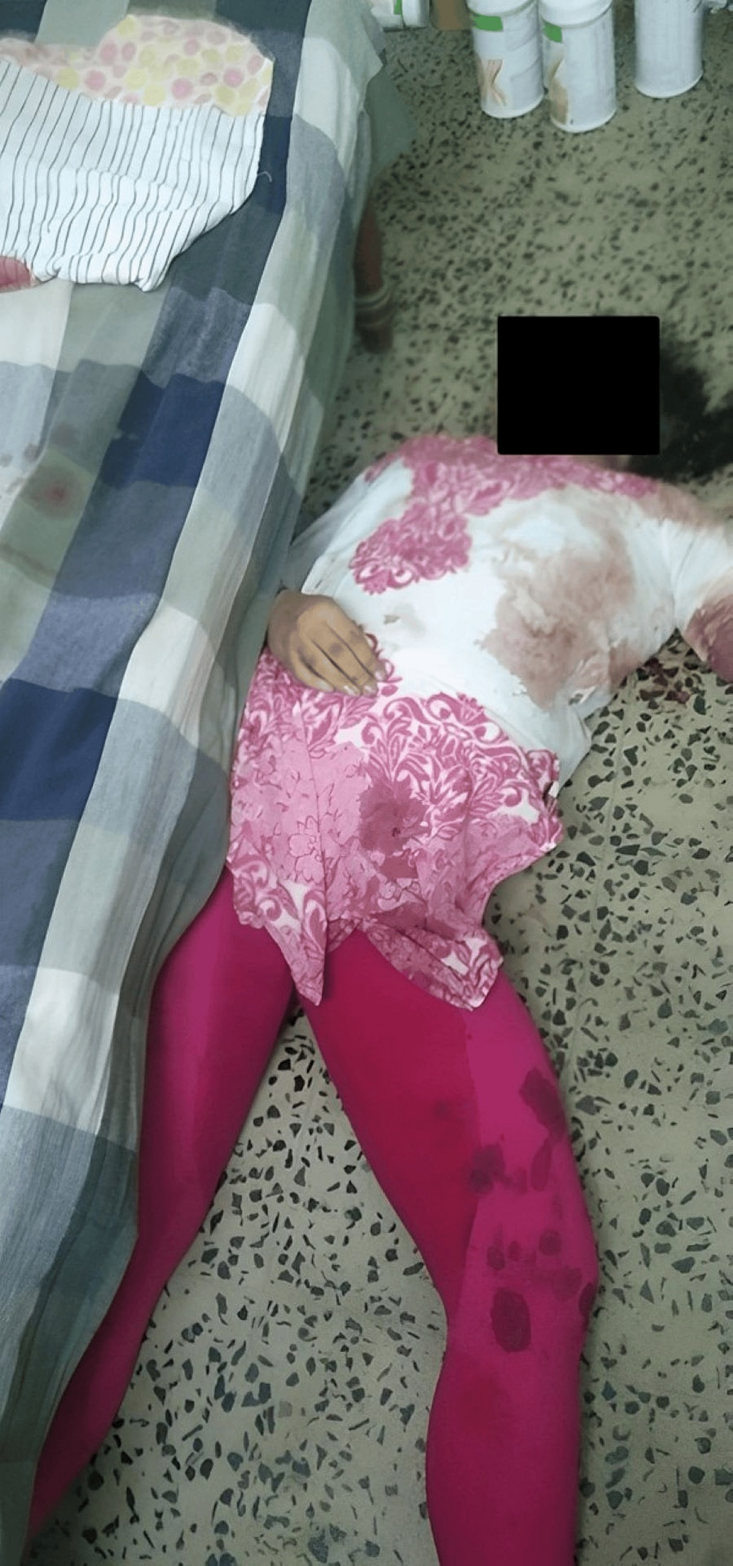
The deceased 41-year-old married woman recovered from the scene of the suicide.

**Figure 2:**
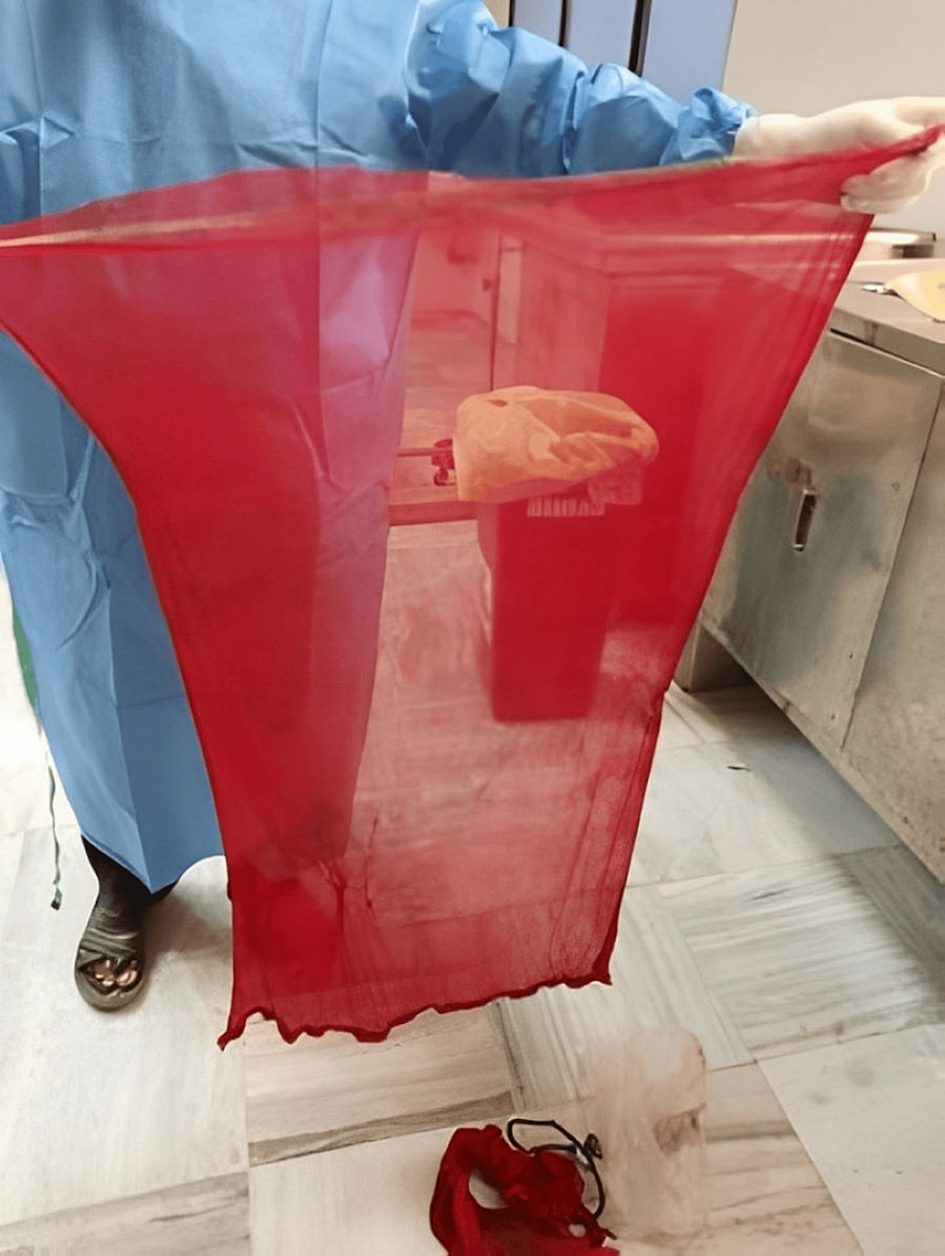
Soft and broad red-colored nylon dupatta used as ligature material with no blood stain.

A 41-year-old married woman (Figure 1) had been working for four years as a teacher in a school near her residence. In the evening after attending school, she worked a part-time job. She had two sons: an 18-year-old college student and a younger son in school. She had been in a disturbed state of mind for a month because of her separation from her husband and financial debt. On July 16, 2023, she was seen going to her room at 5:00 p.m. and did not emerge. She was found dead at 5:30 p.m. Police found her hanging from the ceiling fan by a red-colored dupatta without blood stains (Figure 2). A kitchen knife incision wound was present on her left wrist, with bloodstains on the floor. No suicide note was present at the crime scene. She had no previous history of suicidal attempts or mental illness. A pathologist performed a medicolegal autopsy the next day.

**Figure 3:**
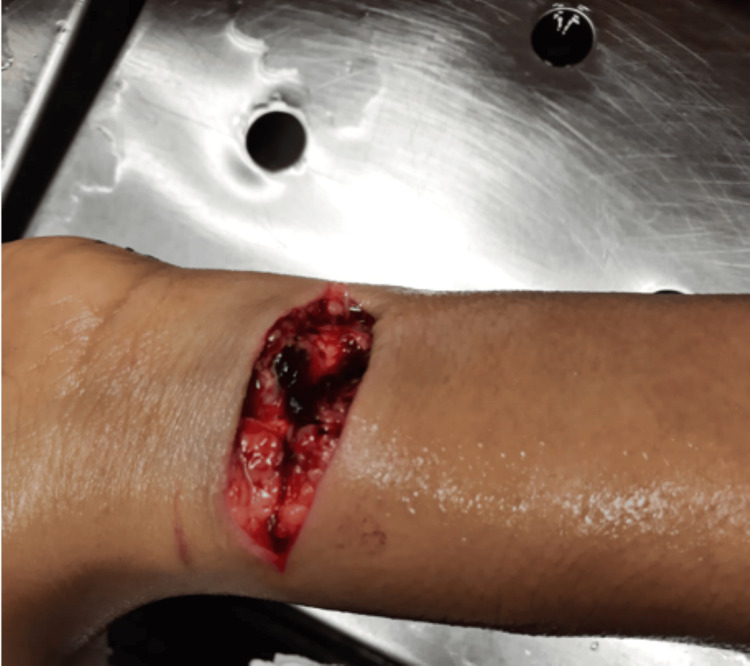
Two incision wounds on the ventral aspect of the left wrist. One appeared to be the hesitation cut, and the other was the fatal cut.

On external examination, the deceased appeared to be a well-built woman. Rigor mortis was present throughout the body, and fixed postmortem hypostasis was observed on the back. Two incision wounds were present on the ventral aspect of the left wrist: one superficial wound measuring 2 cm x 0.1 cm, and another tendon-deep wound measuring 7 cm x 2 cm with underlying superficial blood vessels and subcutaneous tissue incised (Figure 3).

**Figure 4:**
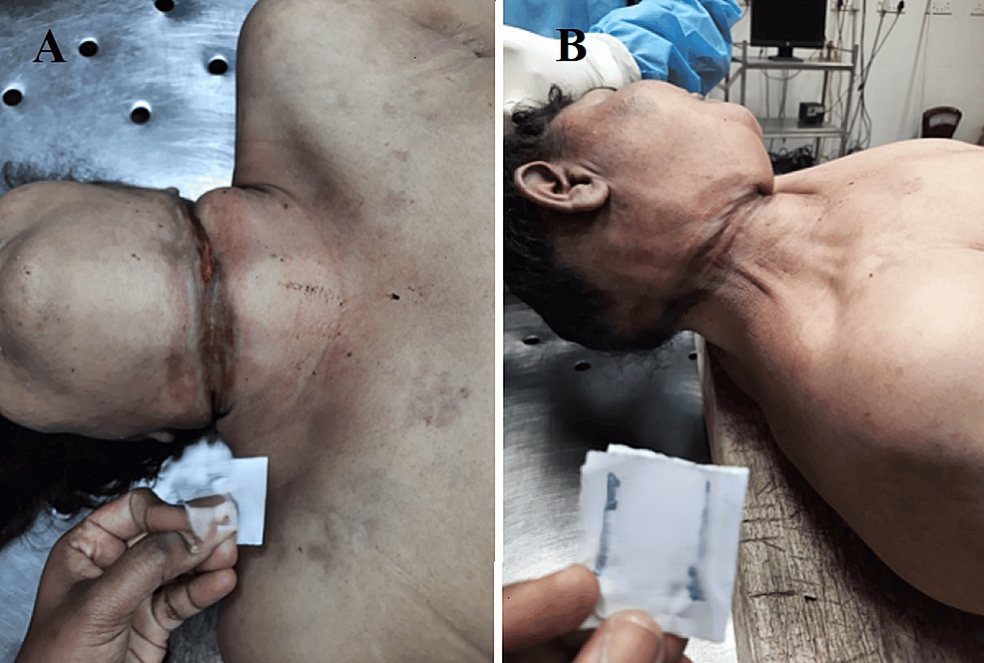
Ligature mark on the anterior and lateral aspects of the neck. A: An oblique, incomplete ligature mark was present over the anterior aspect of the neck above the level of the thyroid cartilage. B: An oblique, incomplete ligature mark was present over the lateral aspect of the neck above the level of the thyroid cartilage.

**Figure 5:**
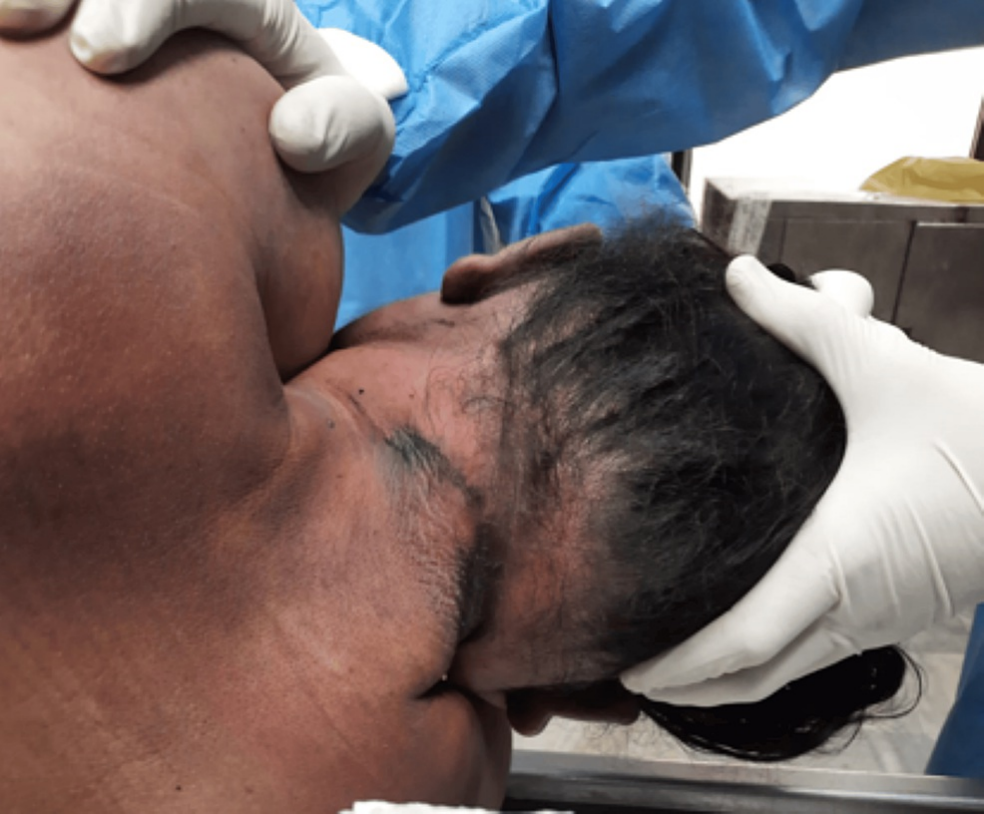
Broad ligature mark at the posterior aspect of the neck with discontinuity.

An incomplete ligature mark was present over the neck above the level of the thyroid cartilage, which was discontinuous and oblique in nature. The total length of the ligature mark was about 26 cm, with a maximum width of 3.5 cm (Figures 4, 5).

Internal examination revealed all neck structures were normal. There was no contusion or bulla at the ligature mark, and the hyoid bone was intact. Both lungs were edematous, and a cut section showed congestion. Petechial hemorrhages were present over the anterior surface of the heart. The stomach was empty and the mucosa was congested. The rest of the organs were congested. All findings were consistent with signs of asphyxia. The autopsy findings indicated that the deceased died of asphyxia and venous congestion due to hanging.

## Discussion

Suicide represents a victim’s decision to end their life. However, complex suicide involves the simultaneous use of two or more methods [1]. A planned complex suicide is rare [4]. When the victim dies by complex suicide, it can be difficult to differentiate between suicide and homicide. Therefore, a detailed police investigation and postmortem examination are essential for a definitive determination.

Petkovic et al. published a case report on complex suicide that described an unusual case involving six methods used by the victim [5]. In our case, we report only two methods.

Our victim was a 41-year-old woman with two incision wounds on her left forearm, one of which was a superficial wound indicating a single hesitation cut, contrary to the usual expectation of multiple, parallel superficial cuts in a suicide attempt. The victim was right-handed, but police found the incision wounds on the left wrist, with a knife on the floor in a pool of blood.

The victim’s past medical history and autopsy findings can help determine whether this was an accident, a homicide, or a suicide. In a homicide, the incisions usually indicate defense-type wounds on the hand and other parts of the body. In a suicide, the incisions are typically multiple and parallel.

Our victim also used hanging as a second method. The pathologist found a ligature mark on her neck above the level of the thyroid cartilage, which was discontinuous and oblique in nature. The ligature mark’s width indicated that the victim used a soft, broad material consistent with a nylon dupatta, which was red in color and without blood stains, as produced by the police during the postmortem. Dissection of the neck revealed that the underlying strap muscles and neck muscles were pale and parchmentized. Based on the inquest report, photographs of the scene of the suicide, and the postmortem findings, we deduced that the victim committed suicide and died of asphyxia and venous congestion due to hanging.

## Conclusions

This case involves a 41-year-old woman who used two methods in a rare instance of complex suicide: incision wounds on her left wrist and hanging. The investigation highlights several key points supporting the determination of suicide. The victim had two incisions on her left forearm: one was a hesitation cut, indicative of a suicide attempt; the other was consistent with self-infliction because she was right-handed. Police found a knife on the floor in a pool of blood, further indicating a self-inflicted injury. The ligature mark on the victim’s neck was discontinuous, oblique, and broad, consistent with a soft material such as the nylon dupatta found at the scene. The pale and parchment-like appearance of the underlying strap and neck muscles supports hanging as the cause of death. The nature of the wounds, the absence of defensive injuries, and findings from the suicide scene and autopsy confirm a self-inflicted death. The evidence indicates the victim died of asphyxia and venous congestion from hanging, supporting the conclusion of suicide.
